# Ethanol Extract of *Cordyceps militaris* Grown on Germinated Soybeans Attenuates Dextran-Sodium-Sulfate- (DSS-) Induced Colitis by Suppressing the Expression of Matrix Metalloproteinases and Inflammatory Mediators

**DOI:** 10.1155/2013/102918

**Published:** 2013-06-12

**Authors:** Dong Ki Park, Hye-Jin Park

**Affiliations:** ^1^Department of Bioscience and Biotechnology, Konkuk University, 1 Hwayang-dong, Gwangjin-gu, Seoul 143-701, Republic of Korea; ^2^Cell Activation Research Institute, Konkuk University, 1 Hwayang-dong, Gwangjin-gu, Seoul 143-701, Republic of Korea

## Abstract

The effect of *Cordyceps militaris* (CM) grown on germinated soybeans (GSC) in the inflammatory bowel disease (IBD) model was studied. To demonstrate the preventive effect of GSC extract in a dextran-sodium-sulfate- (DSS-) induced acute colitis mouse model, GSC was administered 2 days before DSS coadministration. GSC significantly suppressed DSS-induced disease activity index (DAI) as well as histopathological scores, compared to control or CM-treated group. To elucidate the anti-IBD activity of GSC, we checked the level of matrix metalloproteinases (MMPs) and inflammatory mediators. GSC extract decreased the level of MMP-3 and -9 mRNAs and p53 proteins. The level and activity of LPS-induced MMP-9 were reduced in GSC-treated RAW264.7 cells. It also attenuated the level of inducible nitric oxide synthase (iNOS) and tumor necrosis factor- (TNF-) **α** mRNAs both in colon tissue and in macrophage cells. These results suggest that GSC can be applied as a protective agent against IBDs.

## 1. Introduction

Inflammatory bowel diseases (IBDs), including Crohn's disease (CD) and ulcerative colitis (UC), are inflammatory disorders of the gastrointestinal tract caused by genetic and environmental factors. Characteristic symptoms of IBD are accompanied with diarrhea, bloody stools, abdominal pain, and weight loss. Histological characteristics of IBD include crypt abscesses, crypt distortion, ulceration, and infiltration of large numbers of neutrophils, monocytes, and lymphocytes [1].

 The pathogenesis of IBD is still under investigation. The expression of matrix metalloproteinases (MMPs) is upregulated during the pathogenesis of human IBD and experimental colitis. MMPs play a role in the extracellular matrix turnover, having the ability to cleave the majority of extracellular matrix proteins. However, dysregulated expression of MMPs has been shown to have a pathogenic role in a number of diseases, including colorectal cancer, tumor invasion, and inflammatory bowel disease (IBD) [2]. MMP-9 and MMP-3 enzymes are known to degrade a broad range of extracellular matrix components, including collagen I, collagen IV, and proteoglycans [3]. Overproduction of inflammatory mediators, such as tumor necrosis factor-(TNF-*α*) and nitric oxide (NO), is strongly associated with the pathogenesis of IBD. 

Many conventional IBD therapies were mainly focused on downregulating aberrant immune responses and inflammatory signaling events. Currently anti-inflammatory therapy (e.g., 5-aminosalicylic acid and corticosteroids) and some immunomodulators (e.g., azathioprine, 6-mercaptopurine, and cyclosporine) are commonly used for treating acute IBD. However, more than 20% of IBD patients do not respond to these therapies. In addition, steroid therapy brought undesirable side effects, including infertility and developmental disability [4]. Recently natural products and its derived compounds have been spotlighted as novel drug candidates due to their broad spectra of therapeutic effects with low toxicity over the past few decades. Among traditional medicines, *Cordyceps militaris *(CM) (Family: *Clavicipitaceae*) has been widely used in East Asia to treat inflammatory diseases, anemia, asthma, and cancer. Recent studies demonstrated that CM extracts exerted strong anti-inflammatory and anticancer activities [5]. The anti-inflammatory activity of CM involved suppressing expression of inducible nitric oxide synthase (iNOS) and TNF-*α* protein [6]. However, CM is hard to obtain in nature in large amounts due to the high unit cost of production. Many active compounds from soybeans were reported to be effective in preventing various inflammatory diseases and cancers [7]. In this regard, we used the germinated soybeans (GS) (*Glycine max* (L.) Merr.) as culture media for cultivating CM [7]. Previously our group reported phytochemical analysis result of GSC extract by determining total phenolics (24.25 ± 0.63 mg of gallic acid equivalents/g of extract) and flavonoids (4.36 ± 0.01 mg of naringin equivalents/g of extract), which were higher those that of germinated soybeans (GSs). On the basis of the results of the ABTS, DPPH, and FRAP assays, the antioxidant activities of GSC were significantly higher than those of GSs [8, 9]. Our recent studies have demonstrated the antiallergic activities of GSC extract and its novel isoflavonoid compounds* in vivo* and *in vitro *[9].

However, there is no report to address the effect of GSC on the inflammatory bowel disease (IBD) model. In this study, we used a DSS-induced acute colitis mouse model to examine the preventive effect of GSC extract on IBD.

## 2. Materials and Methods

### 2.1. Chemicals and Materials

CM grown on GS (GSC, Kucari 0903) was provided by the Cell Activation Research Institute (CARI, Seoul, Republic of Korea). Male C57BL/6 mice (6 weeks old) were purchased from ORIENT BIO (Seongnam, Republic of Korea). Dextran sulfate sodium salt (DSS) (molecular weight: 35000~50000 daltons) was purchased from MP Biologicals (Santa Ana, CA, USA). Fetal bovine serum, penicillin, and minimum essential medium were purchased from Invitrogen (Carlsbad, CA, USA). Lipopolysaccharide (LPS) was purchased from Sigma-Aldrich (St. Louis, MO, USA). 2,4-Dinitrophenylated-(DNP-) specific IgE, 2,4-dinitrophenylated bovine serum albumin (DNP-BSA), and 1,4-piperazinediethanesulfonic acid (PIPES) from Sigma-Aldrich (St. Louis, MO, USA) were used. Total RNA Isolation kit was purchased from MACHERY-NAGEL Gmbh & Co. (Düren, Germany). The Revertra Ace qPCR RT kit was purchased from Toyobo Biologics Inc. (Osaka, Japan). The QuantiTect SYBR Green polymerase chain reaction (PCR) kit (Qiagen, Valencia, CA, USA). Primers against iNOS (QT00100275), TNF-*α* (QT00104006), and GAPDH (QT01658692) were purchased from Qiagen (Qiagen, Valencia, CA, USA).

### 2.2. Preparation of GSC Extract

The mycelia of CM were grown on GS (*Glycine max* (L.) Merr.) as previously described [7]. An authenticated voucher specimen of CM (Kucari 0906), GS (Kucari 0907), and GSC (Kucari 0903) is deposited in the Herbarium at the College of Bioscience and Biotechnology, Konkuk University (Seoul, Republic of Korea). Each of GSC, GS, and CM was ground to a fine powder with a grinder. The powder was extracted with 80% ethanol (EtOH) for 48 h. The residue was extracted at room temperature and filtered again. The extract was dried by a rotary evaporator under vacuum at 40°C and stored at −20°C until use. GSC, GS, and CM extracts were dissolved in water and used for the animal experiments.

### 2.3. Cell Culture

RAW264.7 macrophages (TIB-71) cells were obtained from the American Type Culture Collection (Manassas, VA, USA). RAW264.7 cells were cultured in DMEM (Invitrogen Co., Carlsbad, CA, USA) supplemented with 1% penicillin and 10% FBS (Gibco BRL, Grand Island, NY, USA). Cells were grown at 37°C with 5% CO_2_ in a humidified incubator.

### 2.4. Experimental Animals

6-week-old C57BL/6 mice were acclimated under controlled specific pathogen-free (SPF) conditions for 1 week prior to the start of the experiment. All mice were housed in the individual cages and fed with standard laboratory chow in an animal room with 12 h light/dark cycles at temperature of 23 ± 2°C. The animal study was performed under institutional guidelines (the Institutional Animal Care and Use Committee (IACUC) at Konkuk University (Seoul, Republic of Korea)). The authorization code number from IACUC is ku09048. 

### 2.5. Experimental Protocol

Acute colitis was induced in C57BL/6 mice (7 weeks old) by adding DSS (MP Biologicals, USA) to drinking tap water at the level of 3% for 7 days as described previously [10]. GSC (500 mg/kg/day) was administered by oral gavage. Prophylactic treatment was defined as the administration of GSC, CM, and GS before DSS treatment. In this experiment, we followed the prophylactic treatment protocol. Mice were randomly assigned to 3 groups (*n* ≥ 15 per group). In Group 1, mice were administered with drinking water for 9 days (*n* ≥ 15 per group); in Group 2, mice were administered with drinking water for 2 days before starting coadministration with 3% DSS (*n* ≥ 15 per group); Group 3, Samples (GSC, CM, and GS) (500 mg/kg/day) were given for 2 days before the coadministration with 3% DSS (*n* ≥ 15 per group).

### 2.6. Evaluation of Disease Activity Index (DAI)

Disease activity index (DAI) was used for evaluation of the grade and extent of intestinal inflammation as previously described [11]. Body weight, stool consistency, and blood in the stool were monitored daily for determination of the DAI.

### 2.7. Assessment of Histological Score

Colon tissue sections were stained with hematoxylin and eosin as previously described [12]. Stained sections were examined by light microscopy (Nikon Co., Japan) (magnifications: 100x and 200x). The histological scoring system was used for evaluating the degree of colitis with H&E images as previously described [6]. 

### 2.8. Measurement of NO

Nitrite concentrations, an indicator of NO production, in RAW264.7 cell culture media were measured as described previously [13]. For these assays, cells (5 × 10^4^ cells/mL) were pretreated in the presence or absence of GSC or CM extract for 1 h before lipopolysaccharide (LPS; 1 *μ*g/mL, Sigma) stimulation for 20 hours. 

### 2.9. Cell Viability

The viability of RAW264.7 cells was determined by the EZ-CyTox kit (Daelillab service Co., Korea) as described previously [6]. RAW264.7 cells (2 × 10^4^ cells/well) were plated on a 96-well plate and incubated in the presence or absence of GSC extracts at concentrations of 10, 100, and 500 *μ*g/mL for 24 h, respectively. A fixed amount (10 *μ*L) of EZ-CyTox reagent was added to each well. After incubation for 2 h at 37°C, absorbance at 450 nm was detected by using an ELISA Multi-Detection Reader (Tecan, Mannedorf, Switzerland).

### 2.10. Immunohistochemistry

The distal colon was dissected and then the longitudinal section (1.5 cm from the anal verge) was prepared. The immunohistochemical staining of colon section (4 *μ*m) was performed using ImmunoCruz ABC staining system (Santa Cruz, CA, USA). Antibodies against inducible nitric oxide synthase (iNOS) (ABCAM, USA, 1 : 300) and p53 (Santa Cruz, CA, USA, 1 : 100) were used for the microscopic analysis. After incubation of primary antibody, the sections were incubated with a biotinylated secondary antibody for 30 min and then with an HRP-streptavidin complex to detect secondary antibody for 30 min. The sections were developed by DAB chromogen kit (Vector laboratories, Burlingame, CA, USA) and were counterstained with 1% methyl green for 1 min.

### 2.11. Real-Time Polymerase Chain Reaction (Real-Time-PCR)

Total RNA was isolated from tissue homogenates as previously described [7]. Amplification reactions were carried out in a total volume (20 *μ*L) of 10 *μ*L containing 2x SYBR Green PCR Master Mix, 2 *μ*L 10x QuantiTect Primer Assay (Qiagen, Valencia, CA, USA), 100 ng cDNA, and 6 *μ*L RNase-free water variable. Real-time-PCR was performed using an ABI500 thermal cycler (Applied Biosystems, Foster City, CA, USA). Data were normalized for the amount of glyceraldehydes-3-phosphated dehydrogenase (GAPDH) mRNA. Levels of TNF-*α*, iNOS, MMP-3, MMP-9, and glyceraldehydes-3-phosphated dehydrogenase mRNAs were measured by Delta Ct value Real-time-PCR. Specific primer sets for TNF-*α* (QT00104006), iNOS (QT00100275), MMP-3 (QT00107751), MMP-9 (QT00108815), and GAPDH (QT01658692) were designed using the Primer Express Program (Applied Biosystems).

### 2.12. Gelatin Zymography

Equal volumes of conditioned medium samples were resolved by electrophoresis on 7.5% SDS-polyacrylamide gels containing 2 mg/mL gelatin. Thereafter, gels were renatured in 2.5% Triton X-100 for 30 min to remove SDS and then incubated with 50 mM Tris-HCl (pH 7.5) containing 10 mM CaCl_2_, 50 mM NaCl, and 0.05% Brj35 for overnight. Gels were stained with 0.25% Coomassie Brilliant Blue, and gelatinolytic activity was quantified using NIH Image1.61 software.

### 2.13. Statistical Analysis

Results are expressed as mean ± SD (*n* ≥ 15 per group). One-way ANOVA was used for assessing significance between control group and sample-treated groups. Statistical analysis was performed using SPSS, version 12 (SPSS Inc., Chicago, IL, USA).

## 3. Results

### 3.1. GSC Prevents DSS-Induced Acute Colitis Symptoms

**Figure 1:**
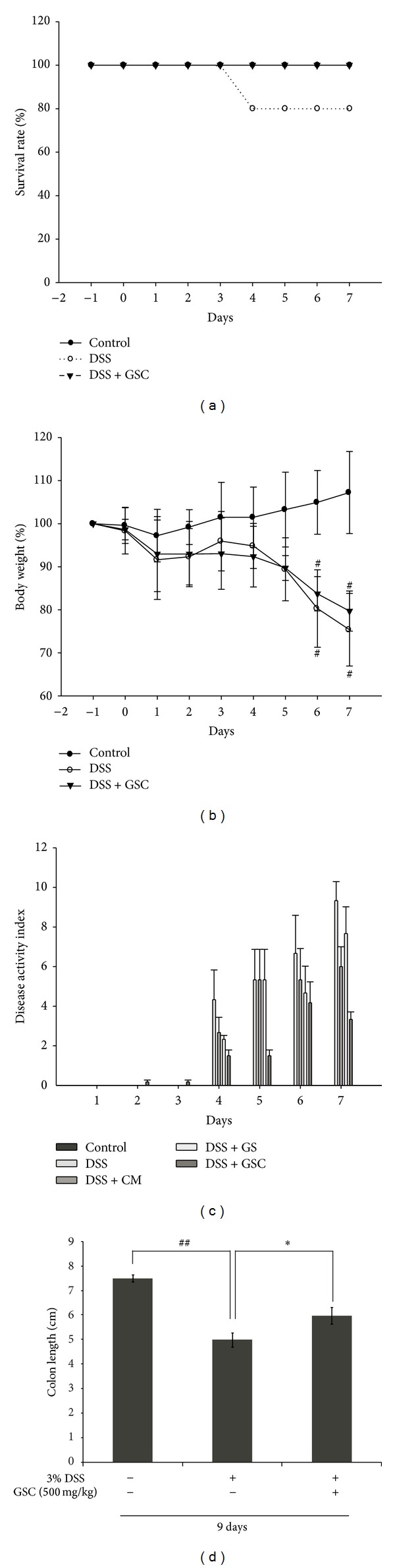
*Cordyceps militaris* grown on germinated soybeans (GSC) extract attenuated the severity of DSS-induced acute colitis. (a) Survival curves of mice administered with the vehicle, DSS, and DSS + GSC extracts. (b) Change of body weight was measured. Statistical significance was assessed compared with control mice (^#^ *P* < 0.01 versus control; *P < 0.01 versus DSS). (c) Disease activity index (DAI) score was monitored daily (*n* ≥ 15 per group). Data are expressed as mean ± standard deviation (SD) (*n* ≥ 15 per group). (d) Colon length of each mouse was measured. (^##^ *P* < 0.001 versus control; *P < 0.01 versus DSS). DSS: dextran sulfate sodium; CM: *Cordyceps militaris*; GS: germinated soybeans; GSC: *Cordyceps militaris* grown on germinated soybeans.

To evaluate the anti-inflammatory effects of GSC, we used mice with DSS-induced acute colitis, which exhibits similar symptoms to the acute phase of human ulcerative colitis. To study the prophylactic effect of GSC, GS, and CM, mice were administered with GSC (500 mg/kg) for 2 days before DSS administration. Mortality was observed on day 6 only in DSS-treated group (Figure 1(a)). At days 8 and 9, GSC extract (500 mg/kg/day) markedly reduced the severity of DSS-induced acute colitis symptoms as evidenced by the decrease of weight loss (*P < 0.01 versus DSS) (Figure 1(b)). In order to determine if GSC extract can attenuate acute colitis symptoms, GSC extract-treated group was compared with CM extract-treated group or germinated soybean extract-treated group. We quantitatively scored clinical symptoms, using the disease activity index (DAI) (e.g., body weight loss, diarrhea, and gross bleeding). An induction in DAI had been observed in DSS-treated mice. DAI score was significantly decreased in GSC extract-treated group compared to CM extract- or GS extract-treated group (Figure 1(c)). Accordingly, we chose the GSC as the test sample. It has been reported that the length of the colon is inversely linked to the severity of DSS-induced acute colitis. We found that the colon of GSC-(500 mg/kg/day) administered mice was significantly longer than that of the DSS-treated group (Figure 1(d)).

### 3.2. GSC Ameliorates Histological Changes

**Figure 2:**
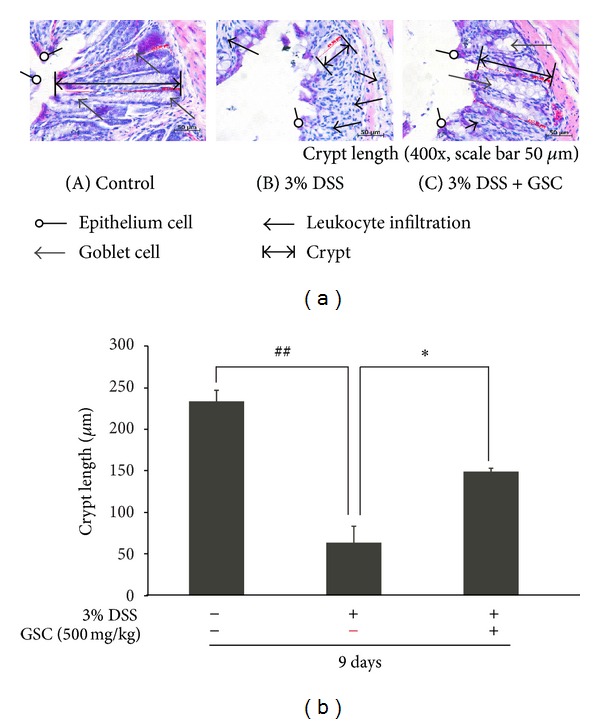
Effect of GSC extract on colonic histological changes and crypt length in mice with DSS-induced colitis. (a) Representative histological findings in mice with dextran-sodium-sulfate- (DSS-) induced colitis. Representative H&E staining data of colon tissue from (A) normal mice administered with drinking water (*n* ≥ 15 per group), (B) mice administered with DSS-induced colitis, and (C) mice coadministered with DSS and GSC extracts (500 mg/kg/day). Open circle arrows indicate epithelium cells. Open triangular arrows indicate goblet cells; closed triangular arrows indicate leukocytes. (b) Crypt length was measured under a light microscope (400x; scale bar = 50 *μ*m) (^##^ *P* < 0.001 versus control; *P < 0.01 versus DSS). Data is expressed as mean ± SD (*n* ≥ 15 per group). DSS: dextran sulfate sodium; GSC: *Cordyceps militaris* grown on germinated soybeans.

We examined the architecture of colonic structure microscopically, using Hematoxylin and Eosin (H&E) staining method. Mice with DSS-induced acute colitis showed loss of the epithelial barrier, significant loss in the number of crypts and goblet cells, and marked infiltration of lymphocytes into the mucosa and submucosa, compared to control group. GSC extract-treated group had the preserved mucosal structure, moderate loss of crypts, epithelial cells, and goblet cells, and less infiltration of inflammatory cells in the colonic tissues compared to the control group (Figure 2(a)). Colonic crypt length of DSS + GSC extract-treated group was longer than that of DSS-treated group (DSS, 63.92 ± 14.47 *μ*m versus DSS + GSC extract, 137.53 ± 19.83 *μ*m, resp.) (Figure 2(b)). Mean histopathology scores were lower in the group fed with GSC extract (4.00 ± 4.41) than those in the DSS-treated group (10.67 ± 1.73) (Table 1). 

### 3.3. GSC Attenuates MMP-3 and MMP-9 mRNA Expressions in Colon Tissue of DSS-Induced Colitis

**Figure 3:**
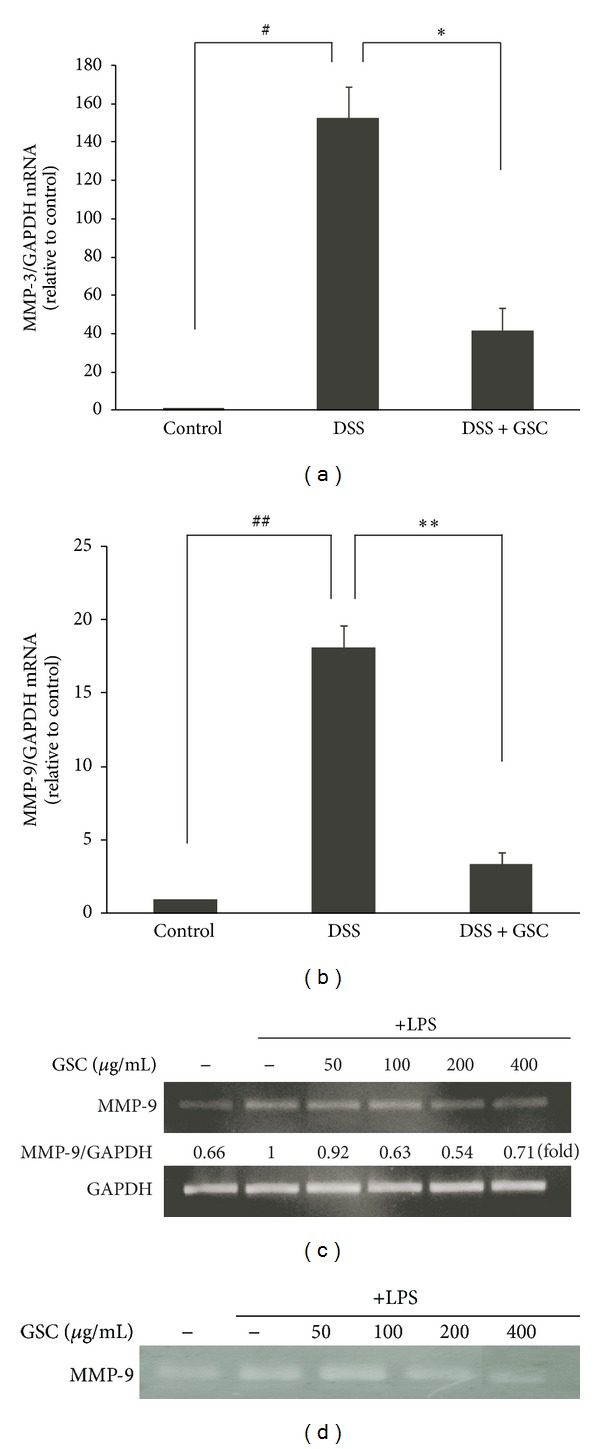
Effect of GSC extract on the mRNA expression of MMP-3 and -9 in the colon tissue of mice with DSS-induced colitis and in LPS-stimulated RAW264.7 macrophages. ((a), (b)) Levels of MMP-3 and -9 mRNA in colon tissue were determined by quantitative ΔΔC_T_ Real-time-PCR, using GAPDH mRNA as the internal control. The graph shown is representative of three independent experiments (^#^ *P* < 0.01, ^##^ *P* < 0.001 versus control; *P < 0.01, **P < 0.001 versus DSS). (c) MMP-9 mRNA levels in LPS-stimulated RAW 264.7 cells were measured by Real-Time-PCR. Cells were incubated for 5 h with 1 *μ*g/mL of LPS in the absence or presence of GSC extract. GSC extract was added 1 h before the incubation with LPS. GAPDH was used as the internal control. Data are expressed as fold change in mRNA transcript levels relative to the LPS-stimulated control. (d) Zymographic image of cell lysates of LPS-stimulated RAW264.7 macrophages after preincubation with GSC extract.

Increased levels of MMP-3 and -9 expressions have been observed in patients with IBD, which presumably have a pathogenic role in elevating proteolysis of the mucosa, ulceration, inflammation, and fistula formation [14]. In IBD animal models, several synthetic, broad spectrum MMP inhibitors significantly diminished disease symptoms compared to placebo-treated controls [15]. Of the MMPs, MMP-9 is a protease that is most abundantly expressed in inflamed tissues of IBD [2]. Consequently we examined the level of MMP mRNA expression in mice with DSS-induced acute colitis after GSC treatment. We found that the levels of MMP-3 and -9 mRNA expressions in colonic tissues of DSS-treated mice were upregulated, while those in GSC-treated mice were decreased (Figures 3(a) and 3(b)). 

Since activated macrophages are known to aggravate the colitis symptoms by inducing the MMP-9 expression [16], we checked whether GSC extract affected MMP-9 mRNA expression in LPS-induced RAW264.7 cells. In concert with *in vivo* data, GSC extract suppressed the level of MMP-9 mRNA expression in LPS-stimulated RAW264.7 cells, compared to control (Figure 3(c)). The gelatinolytic activity of MMP-9 is also decreased after GSC extract treatment (Figure 3(d)). These results suggest that GSC might attenuate DSS-induced colitis in mice by suppressing the level of MMP-3 and -9 mRNAs and the activity of MMP-9 since progression of intestinal inflammation is attributed to these proteinases.

### 3.4. GSC Inhibits TNF-*α* and iNOS mRNA and iNOS Protein Expression in Colonic Tissue of DSS-Induced Colitis

**Figure 4:**
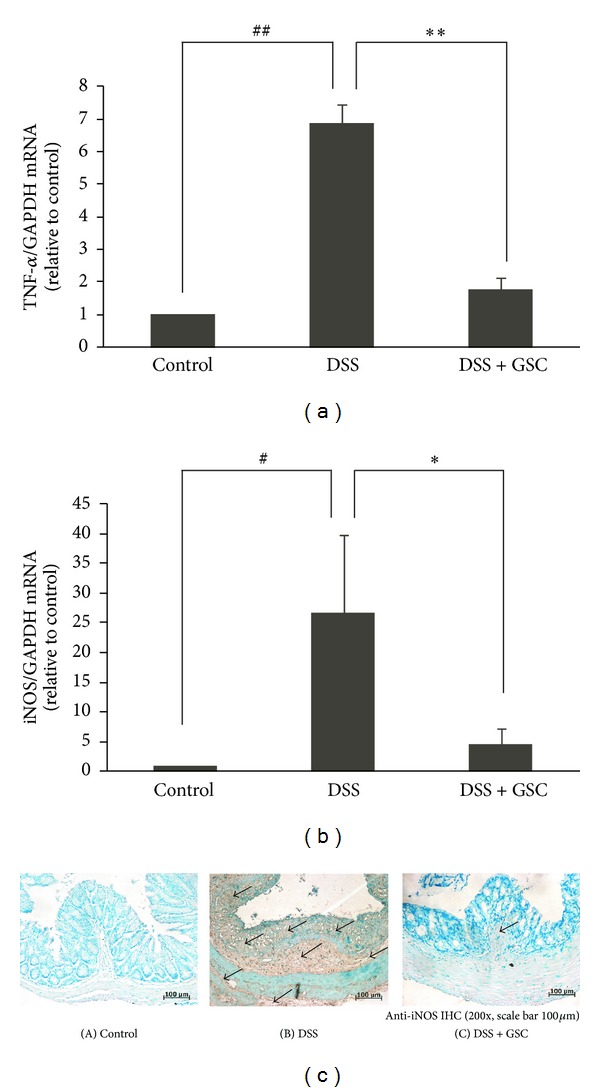
Effect of GSC extract on the levels of TNF-*α* and iNOS mRNA and protein expression in the DSS-treated colon tissue. ((a), (b)) Levels of TNF-*α* and iNOS mRNA in the colon tissue were determined by quantitative ΔΔC_T_ Real-Time-PCR, using GAPDH mRNA as the internal control. The graph shown is representative of three independent experiments (^#^ *P* < 0.05, ^##^ *P* < 0.001 versus control; *P < 0.05, **P < 0.001 versus DSS). (c) Immunostaining of iNOS in the colon using anti-iNOS polyclonal antibody (200x; scale bar = 100 *μ*m) (A) control mice administered with drinking tap water (*n* ≥ 15 per group), (B) DSS-induced mice administered with DSS in drinking tap water, and (C) co-administered with DSS drinking water and GSC extract oral injection (500 mg/kg/day). Open arrows indicate iNOS protein(s).

Colonic injury by DSS administration resulted from an increase of proinflammatory cytokine TNF-*α* [11]. We next measured the levels of TNF-*α* mRNA in colon tissue. GSC extract significantly blocked TNF-*α* mRNA expression in colonic tissues of mice with DSS-induced acute colitis (Figure 4(a)). Nitrosative stress from inducible-NOS-(iNOS-) derived NO contributes to the progression and the pathogenesis of human IBD and experimental colitis. Several synthetic iNOS inhibitors were effective in suppressing DSS-induced colitis symptoms in mice [17]. To elucidate the molecular inhibitory mechanisms of GSC extract against DSS-induced colitis, we measured iNOS mRNA expression. Reduced level of iNOS mRNA expression was observed in the GSC extract-treated group, compared to DSS-treated group (Figure 4(b)). Immunohistochemical analysis result showed that less iNOS protein in epithelium, mucosa, and infiltrating inflammatory cells was expressed in GSC extract-treated group than that in DSS-induced acute colitis group (Figure 4(c)). 

### 3.5. GSC Suppresses TNF-*α* and iNOS mRNA in LPS-Stimulated RAW264.7 Cells

**Figure 5:**
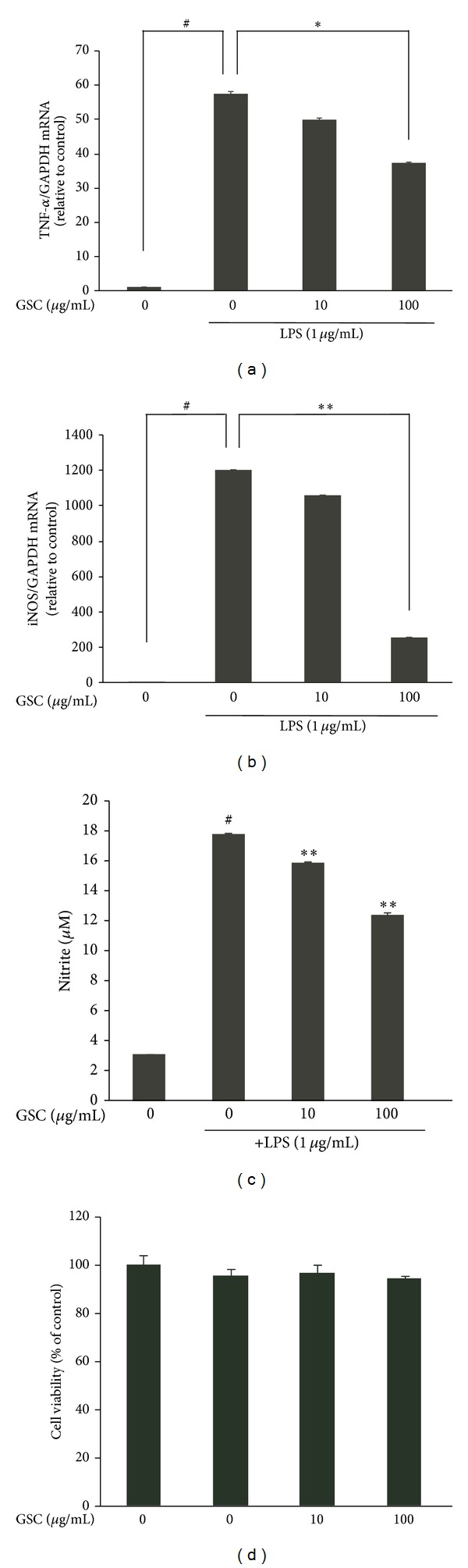
Effect of GSC extract on the level of TNF-*α* and iNOS mRNA expression and NO production in LPS-stimulated RAW264.7 macrophages. ((a), (b)) Levels of TNF-*α* and iNOS mRNA were determined by quantitative ΔΔC_T_ Real-Time-PCR, using GAPDH mRNA as the internal control. The graph shown is representative of three independent experiments (^#^ *P* < 0.05, ^##^ *P* < 0.001 versus control; *P < 0.05, **P < 0.001 versus DSS). (c) NO production in LPS-stimulated RAW264.7 cells. (d) RAW264.7 cell viability. One-way ANOVA was used for comparisons of multiple group means, followed by Dunnett's *t*-test (^#^ *P* < 0.001 versus control; *P < 0.01, **P < 0.001 versus LPS). LPS: lipopolysaccharide. The graph shown is representative of three independent experiments.

Since macrophages are a major source of NO and proinflammatory cytokines, including TNF-*α* [17], we investigate the effect of GSC extract on the TNF-*α* levels and production of NO in lipopolysaccharide- (LPS-) stimulated RAW264.7 cells. The level of TNF-*α* mRNA expression was decreased in GSC-treated RAW264.7 cells, which were stimulated by LPS (Figure 5(a)). GSC extract reduced NO production and iNOS mRNA expression in LPS-stimulated RAW264.7 cells (Figures 5(b) and 5(c)). The cytotoxicity of GSC extract in RAW264.7 cells was evaluated using a CCK-8 assay. The concentrations (0, 10, 100, and 500 mg/mL) of GSC extract did not affect RAW264.7 cell viability (Figure 5(d)). In addition, we found that GSC inhibited NO production stronger than GS or CM (data not shown).

### 3.6. GSC Decreases p53 Expression in Colon Tissue of DSS-Induced Colitis

One form of epithelial cell injury in inflamed colonic mucosa in UC is an apoptotic cell death of these cells [18]. DSS markedly decreased the proliferation of enterocytes in the colon [15]. In addition, aberrant nitrosative stress induces epithelial apoptosis in the colon [15].

**Figure 6:**
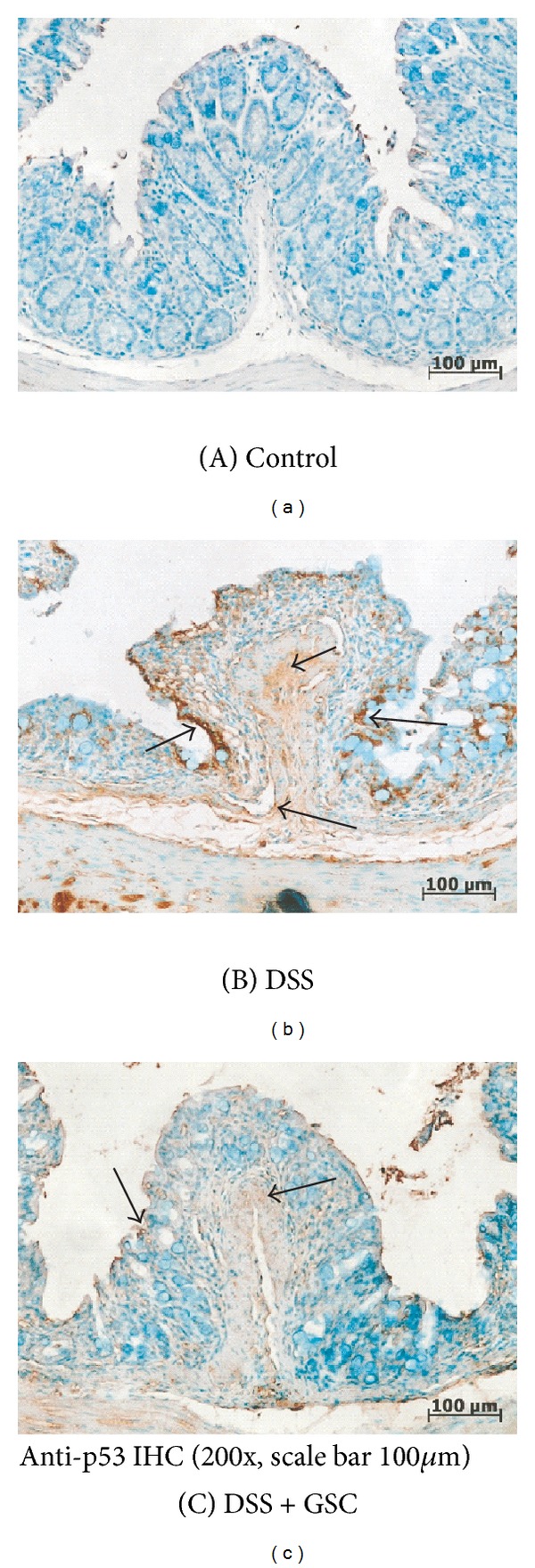
Effect of *Cordyceps militaris* grown on germinated soybeans (GSC) extract on the protein expression of p53 in colon tissue of mice with DSS-induced colitis. Representative immunohistochemical staining for p53 in a colonic section (200x; scale bar = 100 *μ*m) from (A) control mice administered with drinking tap water (*n* ≥ 15 per group), (B) DSS-induced mice administered with DSS in drinking tap water (*n* ≥ 15 per group), and (C) co-administered with DSS drinking water and GSC extract oral injection (500 mg/kg/day) (*n* ≥ 15 per group). Open arrows indicate p53 protein(s).

In control group, p53 protein was occasionally expressed in epithelial cells and crypts. In contrast, the expression of p53 proteins increased dramatically in DSS-administered mice. GSC extract treatment decreased the level of p53 proteins in the colon tissue and preserved the architecture of colonic epithelial cells (Figure 6). It is possible that GSC diminished the apoptotic cell death in the colon by suppressing NO production.

## 4. Discussion

Among IBDs, ulcerative colitis (UC) is a nonspecific inflammatory disease of the large intestine [19]. Currently, anti-inflammatory or immunosuppressive drugs are used for treating UC with side effects, including diarrhea, cramps, and abdominal pain. Novel therapies must be developed for treating the colonic inflammation associated with fewer side effects. Natural products and traditional medicines are nowadays being reevaluated by many researchers for their therapeutic efficacies.

*Cordyceps militaris* (CM), a traditional medicinal mushroom, is cultivated on the dead body of pupa. It has been used from the ancient time in East Asia for treating inflammation, anemia, asthma, and cancer [6]. GSC is *C. militaris* cultivated on the germinated soybeans, not on the pupa. We reported several publications that described the biological activity of additional components of GSC extract that were not found in CM. For instance, novel isoflavonoids were found in GSC [8] and one of them exerted antiallergic activity [9]. However, the anti-inflammatory activity of GSC has not been investigated. In this study, we tested GSC against IBD using DSS-induced animal colitis model. DSS-administered C57BL/6 mice exhibit symptoms similar to those of human ulcerative colitis, such as body weight loss, diarrhea, bloody feces, mucosal ulceration, colonic shortening, crypt abscesses, crypt distortion, and infiltration of neutrophils, monocytes, and lymphocytes [20]. We used 500 mg/kg of GSC EtOH extract (Figure 1). Using dose scaling as advised in FDA guidance, a 60 kg human would have to consume approximately 609 mg per day in order to receive alike benefit. GSC extract was orally given to C57BL/6 mice before DSS administration to evaluate the role of pretreatment with this extract. Then, we assessed body weight loss, stool consistency, stool blood, and DAI score. Histopathological results were consistent with DAI data. The DAI score of GSC extract was lower than that of CM or GS extract. We observed the inflammatory cell infiltration, mucosal erosion, and loss of crypts, epithelial cells, and goblet cells in the colon tissue of DSS-induced colitis mice. GSC extract improved all of these histopathological symptoms. 

Several groups have demonstrated that CM contains numerous components including Myo-inositol, cordycepin, and adenosine, which have anti-inflammatory and immune modulating activities [21]. Metabolomic analysis revealed that novel isoflavone methyl-glycosides (daidzein 7-*O*-*β*-d-glucoside 4′′-*O*-methylate (CDGM), glycitein 7-*O*-*β*-d-glucoside 4′′-*O*-methylate (CGLM), genistein 7-*O*-*β*-d-glucoside 4′′-*O*-methylate (CGNMI), and genistein 4′-*O*-*β*-d-glucoside 4′′-*O*-methylate (CGNMII)) were isolated from GSC, which were neither found in CM nor GS [8, 9]. Recently we demonstrated antiallergic activities of GSC extract and its novel isoflavones identified from GSC on mast cells and on passive cutaneous anaphylaxis model [9]. Isoflavones have various biological properties such as estrogenic, antioxidative, anti-inflammatory, and antiosteoporotic functions [22]. Mast cells are known to play a role in the pathophysiology of IBD, including ulcerative colitis. We assumed that enhanced anti-inflammatory activity of GSC in ulcerative colitis murine model might be due to the presence of these novel compounds, which are not present in either GS or CM. Further studies are needed to elucidate these bioactive components from GSC extract and their anti-inflammatory mechanism against IBD.

MMPs have been demonstrated to be important regulatory molecules in the pathogenesis of inflammatory diseases and cancer. MMPs can degrade to a broad range of extracellular matrix components, including collagen I, collagen type IV, and proteoglycans. In addition, MMP-3 can activate other MMPs [14]. The level of MMPs is closely correlated with the severity of acute or chronic inflammatory bowel diseases (IBDs). Elevated levels of MMP-2, -3, -7, and -9 mRNA and proteins were observed in mice with DSS-induced colitis [15]. Several groups have reported that synthetic inhibitors that suppress mRNA expression and/or enzymatic activity of MMPs are effective in treating inflammatory bowel diseases. We therefore tested MMP-3 and MMP-9 expression levels in colonic mucosa using Real-time-PCR. Our results showed that GSC extracts inhibited the upregulated level of MMP-3 and MMP-9 mRNAs in mice with DSS-induced acute colitis. These findings indicate that GSC extract attenuates the symptom of acute colitis via inhibition of MMP expression and activity. 

Oxidative stress is a major cause of tissue damage and inflammation [23]. Sustained high NO production, especially when mediated by iNOS in the colon, plays a role in the pathology of IBD. Several studies have shown that *Cordyceps militaris* extract suppresses iNOS expression and NO production in macrophages [11]. The previous study demonstrated that the severity of DSS-induced colitis was significantly attenuated in iNOS knockout and in specific iNOS inhibitor-treated animals [24]. Our data demonstrated that GSC extracts decreased the level of iNOS mRNA expression in colon tissues and in macrophages. Presumably, GSC extracts may act as an iNOS inhibitor so that it protects the colon from DSS-induced tissue injury and inflammation by reducing nitrosative stress. Immunohistochemical analysis result showed that less iNOS protein was expressed in epithelium, mucosa, and infiltrating inflammatory cells of the colon tissue in GSC extract-treated group than that in DSS-induced acute colitis group. It was reported that the production of proinflammatory cytokines including TNF-*α* was significantly increased in DSS-administered mice. TNF-*α* damages the epithelial barrier of colon and induces apoptotic death of colon epithelial cells [25]. GSC extract significantly attenuated the level of TNF-*α* mRNA expression in the colonic tissues of mice with DSS-induced acute colitis and LPS-stimulated macrophage cells.

The epithelial cell injury in inflamed colonic mucosa in IBD is due to the apoptotic cell death [26]. p53 is one of the major regulators in apoptosis and its protein level increases in the colon mucosa of IBD [26]. Increased apoptosis in DSS-induced colitis may bring a destruction of the epithelial barrier function and facilitate the mucosal invasion of harmful intestinal bacteria, which leads to the chronic phase of colitis. Here, we observed that less p53 protein was expressed in epithelial cells and crypts of the colon tissue in GSC extract-treated group than that in DSS-treated group. In this study we investigated whether GSC treatment modulates the acute colitis in an experimental model. However, our study lacks demonstrating the effect of GSC in human. The next aim is to obtain the human clinical data of GSC against IBDs. Also identification of compounds from GSC responsible for anti-IBD activities needs to be done. Further studies will explore the effect of compounds, including novel isoflavones from GSC in acute colitis model.

In conclusion, the results showed GSC significantly reduced DSS-induced colitis symptoms through preventing body weight loss and colon shortness and decreasing the DAI scores, compared to GS- or CM-treated group. The protective effects of GSC extract may be attributed to the significant reduction in the level of MMPs and p53 proteins as well as the levels of NO and TNF-*α* expressions. From these results, GSC extract might be applied as an anti-inflammatory agent for preventing inflammatory bowel diseases (IBDs).

## Figures and Tables

**Table 1:** Histological score of DSS-induced colitis in GSC-treated mice.

	Control	3% DSS	3% DSS + GSC
Loss of epithelium	0.67 ± 0.58	2.67 ± 0.58^a^	0.67 ± 1.15^d^
Loss of crypt	0.33 ± 0.58	2.67 ± 0.58^b^	1.33 ± 1.53^c^
Loss of goblet cells	0.00 ± 0.00	2.33 ± 0.58^b^	0.67 ± 1.15^d^
Infiltration of inflammatory cells	0.00 ± 0.00	3.00 ± 0.00^b^	1.33 ± 0.58^d^

Total	1.00 ± 1.16	10.67 ± 1.73^b^	4.00 ± 4.41^e^

Each value represents the mean ± S.E.M. of *n* ≥ 15 animals.

^a^ *P* < 0.01, ^b^ *P* < 0.001 versus Control; ^c^ *P* < 0.05, ^d^ *P* < 0.01, and ^e^ *P* < 0.001 versus DSS.

DSS: dextran sulfate sodium; GSC: *Cordyceps  militaris* grown on germinated soybeans.

3% DSS and GSC extract (500 mg/kg).
